# Masturbation parameters: their relation to sexual arousal in young people who engage in same-sex relationships

**DOI:** 10.3389/fpsyg.2025.1544691

**Published:** 2025-03-19

**Authors:** Gracia M. Sánchez-Pérez, Reina Granados, Pablo Mangas, Juan Carlos Sierra

**Affiliations:** ^1^Mind, Brain, and Behavior Research Center, University of Granada, Granada, Spain; ^2^Department of Nursing, Health Sciences Faculty, University of Granada, Granada, Spain

**Keywords:** masturbation parameters, same-sex relationships, sexual excitation, subjective sexual arousal, genital response

## Abstract

**Background:**

Interest in masturbation in sexual orientation and gender diversity research are rather limited. Extending this research field to include this type of population by considering different masturbation parameters is necessary. In this respect, various masturbation parameters (i.e., negative attitudes toward masturbation, solitary sexual desire, current masturbation frequency, subjective orgasm experience) were validated in a laboratory study with different measures of sexual arousal in persons who engage in same-sex relationships.

**Aim:**

Our main aim was to provide evidence to support the validity of the different masturbation parameters in young people who engage in same-sex relationships. The association between masturbation parameters and various sexual arousal measures (genital response, rating of sexual arousal and rating of genital sensations) was analyzed.

**Methods:**

During a lab task, 72 young adults who engaged in same-sex relationships (36 women, 36 men; age range: 18–32 years) watched content-neutral and sexually explicit films. They included scenes of self-exploration and solitary masturbation behaviors performed by individuals of the same sex as the participants. Negative attitudes toward masturbation, solitary sexual desire, current masturbation frequency, dimensions of subjective orgasm experience in the solitary masturbation context (i.e., affective, sensory, intimacy, rewards), propensity for sexual excitation, genital response (i.e., penile circumference and vaginal pulse amplitude), rating of sexual arousal and rating of genital sensations were assessed. Regression models were conducted to explain the arousal measures with masturbation parameters.

**Results:**

In women, the intimacy dimension of the subjective orgasm experience in masturbation (*β* = 0.42, *p* = 0.007) and solitary sexual desire (*β* = 0.32, *p* = 0.040) predicted the rating of sexual arousal by explaining 24.1% of its variance. Conversely for men, the rewards dimension of the subjective orgasm experience in masturbation (*β* = 0.40, *p* = 0.016) significantly predicted genital response and explained 13.4% of its variance.

**Conclusion:**

Our findings validate some examined masturbation parameters (specifically solitary sexual desire and subjective orgasm experience) in young adults who engage in same-sex relationships. Gender differences in the masturbation parameters were observed for the relevance of these masturbation parameters for explaining sexual arousal. These findings support the relation between masturbation and sexual function.

## 1 Introduction

The study of sexuality has predominantly focused on heterosexual populations (Serrano-Amaya and Ríos-González, 2019). Lack of research into sexual orientation and gender diversity (SOGD) has limited advances being made in sexual health in this population (Mijas et al., 2021). As masturbation is a behavior associated with different sexual health indicators (e.g., sexual satisfaction; Cervilla et al., 2024a), it is important to focus its study on SOGD. However, masturbation research is still limited and usually focuses only on analyzing its frequency (Cervilla et al., 2024a; Fischer et al., 2022). Recently, Cervilla and Sierra (2022) proposed different parameters to examine the study of this behavior in-depth: negative attitudes, solitary sexual desire, current frequency and intensity of subjective orgasm experience. However, these have been examined together only in heterosexual populations (Cervilla and Sierra, 2022; Sierra et al., 2023).

Sexual attitudes, which are understood as beliefs that play an important role in emitting favorable or unfavorable responses toward sexual stimuli, influence the modulation of sexual response (Sierra et al., 2021). It has been observed that negative attitudes toward masturbation are associated with negative sexual experiences (Hogarth and Ingham, 2009). In a way, negative attitudes toward masturbation are linked with lesser subjective sexual arousal in response to erotic visual stimuli (Mosher and Abramson, 1977), and with more difficulties with erection (Cervilla et al., 2021; Sierra et al., 2021) and vaginal lubrication (Cervilla et al., 2021). In contrast, positive attitudes toward masturbation are associated with greater pelvic vasocongestion in response to sexual stimuli (Abramson et al., 1981).

Solitary sexual desire refers to the interest in or willingness to engage in sexual activities with oneself, which may imply the desire to not share sexual experiences with others (Spector et al., 1996). Those who exhibit high solitary sexual desire levels report better sexual functioning (Arcos-Romero et al., 2022; Cervilla and Sierra, 2022; Sierra et al., 2023). Solitary sexual desire has been positively associated with sexual arousal (Santos-Iglesias et al., 2013), propensity for sexual excitation (Cervilla et al., 2023; Moyano and Sierra, 2014; Peixoto et al., 2018; Vallejo-Medina et al., 2020), and women’s genital response toward sexual stimuli (Cervilla et al., 2023).

For masturbation frequency, higher frequency in women is associated with greater ease of sexual arousal (Carvalheira and Leal, 2013). Moreover, in both men and women, higher masturbation frequency is linked with greater sexual arousal (Walton and Bhullar, 2018) and subjective arousal in response to sexual stimuli (Mosher and Abramson, 1977). Therefore, masturbation plays an important role in the awareness of the physiological changes that accompany sexual arousal (Hoon, 1983).

The subjective orgasm experience (i.e., its perception and evaluation; Arcos-Romero and Sierra, 2018) has been described in both the sexual relationships (Arcos-Romero et al., 2018, 2019) and solitary masturbation (Cervilla et al., 2022; Cervilla et al., 2024b) contexts from a multidimensional perspective. It comprises the affective (i.e., feelings experienced during orgasm; e.g., “exciting”), sensory (i.e., physiological sensations; e.g., “throbbing”), intimacy (i.e., the intimate aspect of orgasm; e.g., “tender”), and rewards (i.e., rewarding effects; e.g., “relaxing”) dimensions. Greater intensity of subjective orgasm experience in masturbation is associated with better sexual functioning (Cervilla et al., 2024b; Cervilla and Sierra, 2022). Cervilla et al. (2024b) observed more specifically: in men a positive association between the rewards dimension of orgasm in masturbation and propensity for sexual excitation, and between the intimacy dimension and the rating of sexual arousal; in women a positive association between the sensory dimension and the rating of sexual arousal. Furthermore, Cervilla et al. (2022) found positive correlations between all the orgasm dimensions in masturbation and sexual arousal.

Considering the relevance of including different masturbation parameters beyond its frequency, the importance of analyzing their relation with other sexual functioning measures (Cervilla et al., 2024a), and the need to focus research on SOGD (Pollit et al., 2022), in lesbians, gays and bisexuals (LGB), this study aims to provide evidence for the validity of the masturbation parameters (i.e., negative attitudes, solitary sexual desire, current frequency, intensity of the subjective orgasm experience) through their relation with psychophysiological measures (i.e., genital response) and self-reported sexual arousal (i.e., rating of sexual arousal and genital sensations). The recording of the psychophysiological responses related to sexual arousal in a laboratory context, and their subsequent relation with different sexuality dimensions, constitute a source of validity evidence for them (Álvarez-Muelas and Sierra, 2023).

Based on previous evidence, it is hypothesized that negative attitudes toward masturbation in a negative sense (Abramson et al., 1981; Mosher and Abramson, 1977), and solitary sexual desire (Cervilla et al., 2023), current masturbation frequency (Mosher and Abramson, 1977) and intensity of subjective orgasm experience in masturbation (Cervilla et al., 2024b) in a positive sense, will explain a significant percentage of sexual arousal, specifically genital response (i.e., penile circumference or vaginal pulse amplitude), and the rating of sexual arousal and genital sensations.

## 2 Method

### 2.1 Participants

Seventy-two young cisgender adults participated (36 women and 36 men) aged 18 to 32 years (*M*_women_ = 22.08, *SD* = 2.92; *M*_men_ = 23.61, *SD* = 3.56; *t* = 1.99, *p* = 0.510). The inclusion criteria were (a) aged 18 years or older (b) having same-sex sexual relationships exclusively or predominantly, and (c) having masturbated within the last 3 months before the assessment. The exclusion criteria were (a) having a medical condition that affects sexual responsiveness, (b) being treated for any sexual dysfunction, (c) having psychological disorders, (d) taking medication that could alter sexual responsiveness (e.g., antidepressants or anxiolytics), (e) drugs/alcohol abuse and (f) having a history of sexual abuse and/or victimization. All the participants reported being at university.

Running a power analysis using G*Power (Faul et al., 2007), with an alpha level of 0.05, power = 0.80, mean effect size (Cohen’s *d* = 0.55) and 8 predictors, indicated a sample size of at least 36 participants was required for the regression models.

### 2.2 Measures and materials

Sociodemographic and Sexual History Questionnaire. It includes questions on sex, gender, age, nationality, level of education, type of sexual relationships (with people of the same or different sex), masturbation behavior, medical/psychological/sexual problems, pharmacological treatment, drug/alcohol use, and sexual victimization history.

The Spanish version of the Negative Attitudes Toward Masturbation Inventory (Mosher, 2011b) by Cervilla et al. (2021). It assesses negative attitudes toward masturbation with 10 items (e.g., “I feel guilty about masturbating”) answered on a 5-point Likert scale from 1 (*not at all true*) to 5 (*extremely true*). Higher scores indicate more negative attitudes. Consistency reliability was 0.95 and it provided adequate validity evidence (Cervilla et al., 2021). The Cronbach’s alpha coefficient obtained in this study was 0.62.

The Solitary Sexual Desire Subscale of the Spanish version of the Sexual Desire Inventory (Spector et al., 1996) by Moyano et al. (2017). It assesses solitary sexual desire with four items (e.g., “Compared to other people of your age and sex, how would you rate your desire to behave sexually by yourself?”) using different Likert-type scales (e.g., 0 = *not at all* to 7 = *more than once a day*). Higher scores indicate greater solitary sexual desire. Internal consistency reliability was above 0.90 and its measures provided adequate validity evidence (Moyano et al., 2017). Cronbach’s alpha coefficient obtained in this study was 0.84.

The Spanish version of the Orgasm Rating Scale (Mah and Binik, 2011) validated in the masturbation context by Cervilla et al. (2022). The subjective orgasm experience obtained through masturbation is assessed with 25 adjectives distributed in four factors, namely Affective (i.e., feelings experienced during orgasm; e.g., “fulfilling”), Sensory (i.e., physiological sensations; e.g., “quivering”), Intimacy (i.e., intimate aspect of orgasm; e.g., “close”) and Rewards (i.e., rewarding effects; e.g., “shooting”), on a 6-point Likert scale from 0 (*does not describe it at all*) to 5 (*describes it perfectly*). Higher scores indicate greater intensity in the subjective orgasm experience. Internal consistency reliability ranged from 0.71 (Intimacy) to 0.95 (Sensory), and its measures showed adequate validity evidence (Cervilla et al., 2022). In this study, Cronbach’s alpha coefficients were 0.86 for Affective, 0.92 for Sensory, 0.73 for Intimacy and 0.73 for Rewards.

The Spanish version of the Sexual Inhibition/Excitation Scales-Short Form (Carpenter et al., 2011) by Moyano and Sierra (2014). It assesses propensity for sexual excitation/inhibition by means of 14 items distributed on three subscales: Sexual Excitation (SES; e.g., “When I start fantasizing about sex, I quickly become sexually aroused”), Sexual Inhibition due to Threat of Performance Failure (SIS1; “I cannot get aroused unless I focus exclusively on sexual stimulation”) and Sexual Inhibition due to Threat of Performance Consequences (SIS2; e.g., “If I am having sex in a secluded, outdoor place and I think that someone is nearby, I am not likely to get very aroused”) on a 4-point Likert-type scale from 1 (*strongly agree*) to 4 (*strongly disagree*). Responses were reversed for better interpretation, with higher scores indicating more propensity for sexual excitation/inhibition. It presented adequate validity evidence (Sierra et al., 2019) and adequate internal consistency with coefficients ranging from 0.66 to 0.85 (Sierra et al., 2024). In this study, only the SES scale was considered, whose internal consistency reliability was 0.67.

The Spanish version of the Rating of Sexual Arousal (Mosher, 2011a) by Sierra et al. (2017). It assesses sexual arousal intensity in a given situation using five items (sexual arousal, genital sensations, sexual warmth, non-genital physical sensations and sexual absorption) answered on a Likert scale from 1 (*no arousal at all*) to 7 (*extremely sexually stimulated*). It had adequate internal consistency values (*α* = 0.90) and good validity evidence (Sierra et al., 2019). In this study, a Cronbach’s alpha coefficient of 0.91 was obtained.

The Spanish version of the Rating of Genital Sensations (Mosher, 2011a) adapted by Sierra et al. (2017). It assesses the level of genital sensations to a sexual stimulus using a single item with 11 response options in increasing order of sexual arousal from *No genital sensations* to *Multiple orgasms: repeated orgasmic release in a single sexual episode*. Its measures presented adequate validity evidence (Sierra et al., 2019; Sierra et al., 2017).

The Biopac Model MP150 Polygrah with 16 Channels (Biopac Systems Inc., Goleta, CA, USA) with the AcqKnowledge 5.0. software. It enabled genital response recording with a penile pletysmograph module (Biopac amplifier DA100C and indium/gallium sensors) and a vaginal photoplethysmography module (Biopac amplifier PPG100C and vaginal transducers). The measurements obtained by these devices are the change produced in penile circumference (millimeters) while erection occurs and the vaginal pulse amplitude (volts) due to vasocongestion produced during sexual arousal.

Visual stimuli. Three-minute videos with neutral and sexually explicit content showing men and women masturbating alone to reach orgasms. Visual stimuli with sexual content were previously validated in a pilot study with similar participants to ensure their ability to elicit sexual arousal.

### 2.3 Procedure

By means of posters, social media posts and mailing lists, young adults were invited to participate in the study, which consisted of two phases. In the first phase, interested volunteers accessed an online survey that assessed the variables related to the inclusion criteria. At this point, participants gave their informed consent and provided their email address and telephone number to be contacted for the second study phase. The participants who met the inclusion criteria were scheduled for an appointment in the Human Sexuality Laboratory. Prior to the appointment, participants were sent a new informed consent form and asked to abstain from alcohol, caffeine or any stimulant drinks and sexual activity for 24 h before the study. To avoid variations associated with the menstrual cycle, women were called to the laboratory between days 14 and 28 of their menstrual cycle (Suschinsky et al., 2014).

In the second study phase, in the sexuality laboratory participants signed another informed consent form that guaranteed the anonymity and confidentiality of their data. The researcher then explained the experimental procedure and showed them how to put on the genital response sensors. After providing an explanation, the researcher went to the control room to monitor the stability of the genital response recordings. With the recording devices in place, participants waited 5 minutes for videos projection to get used to the room.

The experimental task consisted of watching two blocks of videos (neutral video 1 + sexual video 1, and neutral video 2 + sexual video 2) whose sexual content corresponded to participants’ sexual orientation (i.e., men masturbating for the men participants and women masturbating for the women participants). Meanwhile, the genital response, which was calculated by the difference between the scores of the sexually explicit stimulus and the neutral stimulus, was recorded, following the indications of previous studies (Cervilla et al., 2024b; Mangas et al., 2024b). Video sequences were counterbalanced to control for order effects. Participants were randomly assigned to view one of two sequences: Sequence A (neutral video 1 + sexual video 1, and neutral video 2 + sexual video 2) or Sequence B (neutral video 2 + sexual video 2, and neutral video 1 + sexual video 1). At the end of each video, participants completed the Rating of Sexual Arousal and Rating of Genital Sensations scales.

All the self-reports, and the data collected from each participant in all the phases, were assigned an alphanumeric code as participant identifiers to always ensure participants’ anonymity.

### 2.4 Data analysis

First by the *t*-test, differences by gender in the variables under study were examined. Next partial correlations were calculated by controlling for propensity for sexual excitation between the masturbation parameters (i.e., negative attitudes toward masturbation, solitary sexual desire, current frequency, subjective orgasm experience dimensions) and genital response, the rating of sexual arousal and the rating of genital sensations.

Finally, to determine the explanatory capacity of the masturbation parameters on the sexual arousal measures, regression models were conducted by following the stepwise method. The predictor variables were divided into two blocks: (1) propensity for sexual excitation and (2) negative attitudes toward masturbation, solitary sexual desire, current masturbation frequency and subjective orgasm experience dimensions in masturbation.

## 3 Results

### 3.1 Comparisons between men and women

As a preliminary step to the study objective, we examined the differences between men and women in the study variables. Table 1 shows the significant differences in negative attitudes toward masturbation (*t* = 2.43, *p* = 0.018, Cohen’s *d* = 0.58), current masturbation frequency (*t* = 3.97, *p* < 0.001, Cohen’s *d* = 0.94) and the rewards dimension of subjective orgasm experience (*t* = 2.10, *p* = 0.04, Cohen’s *d* = 0.50), with the first one having higher scores in all cases.

**Table 1:** Mean score, standard deviation and comparison of the study variables between men and women.

Variables	Women (*n* = 36)	Men (*n* = 36)			
	*M* (*SD*)	*M* (*SD*)	*t*	*p*	Cohen’s *d*
Negative attitudes toward masturbation	10.44 (1.05)	11.47 (2.30)	2.43	0.018	0.58
Solitary sexual desire	21.39 (5.60)	23.56 (3.56)	1.96	0.055	-
Current masturbation frequency	4.44 (0.99)	5.36 (0.96)	3.97	<0.001	0.94
Affective dimension of subjective orgasm experience	28.22 (5.21)	28.94 (4.96)	0.60	0.550	-
Sensory dimension of subjective orgasm experience	42.65 (14.30)	39.32 (12.19)	−1.07	0.291	-
Intimacy dimension of subjective orgasm experience	9.03 (3.66)	8.64 (3.42)	−0.47	0.643	-
Rewards dimension of subjective orgasm experience	12.75 (3.18)	14.17 (2.51)	2.10	0.040	0.50
Propensity for sexual excitation	16.08 (2.89)	16.94 (2.29)	1.40	0.166	-
Rating of sexual arousal	18.75 (5.24)	19.60 (5.79)	0.65	0.517	-
Rating of genital sensations	3.62 (1.26)	3.92 (1.48)	0.90	0.370	-

*t, Student’s t; M, mean; SD, standard deviation.*

### 3.2 Partial correlations

As shown in Table 2, in women the affective (*r* = 0.38, *p* < 0.05), sensory (*r* = 0.34, *p* < 0.05), and intimacy (*r* = 0.52, *p* < 0.01) dimensions of subjective orgasm experience in masturbation were significantly related to the rating of sexual arousal. In men, the affective (*r* = 0.34, *p* < 0.05) and rewards (*r* = 0.40, *p* < 0.05) dimensions of subjective orgasm experience were significantly related to genital response.

**Table 2:** Partial correlations controlling propensity for sexual excitation between study variables.

	Women			Men		
Variables	Genital response	Rating of sexual arousal	Rating of genital sensations	Genital response	Rating of sexual arousal	Rating of genital sensations
Negative attitudes toward masturbation	−0.09	0.32	0.30	−0.24	0.12	0.19
Solitary sexual desire	−0.22	0.31	0.15	0.08	−0.04	−0.07
Current masturbation frequency	−0.22	0.24	0.09	0.10	0.03	0.10
Affective dimension of subjective orgasm experience	−0.11	0.38*	0.22	0.34*	−0.09	−0.22
Sensory dimension of subjective orgasm experience	−0.10	0.34*	0.13	0.33	−0.11	−0.24
Intimacy dimension of subjective orgasm experience	−0.23	0.52**	0.05	0.10	−0.04	−0.17
Rewards dimension of subjective orgasm experience	−0.27	0.14	−0.13	0.40*	0.12	−0.19

** *p* < 0.01, * *p* < 0.05.

### 3.3 Regression models

For women, the rating of sexual arousal was significantly related to the intimacy dimension of the subjective orgasm experience in masturbation (*β* = 0.42, *p* < 0.01) and solitary sexual desire (*β* = 0.32, *p* < 0.05). These variables explained 24.1% of the variance in the rating of sexual arousal (*F*_(2, 33)_ = 6.56, *p* < 0.01) (Table 3).

**Table 3:** Multiple regression models for sexual arousal measures.

	Predictors	*B*	SE	*β*	95% *CI*	*t*	*p*	*R* ^2^	VIF
Women									
**Rating of sexual arousal**									
1								0.161	
	Intimacy dimension of subjective orgasm experience	0.62	0.22	0.43	0.17, 1.07	2.78	0.009		1.000
2								0.241	
	Intimacy dimension of subjective orgasm experience	0.61	0.21	0.42	0.18, 1.04	2.87	0.007		1.001
	Solitary sexual desire	0.30	0.14	0.32	0.02, 0.58	2.14	0.040		1.001
Men									
**Genital response**									
1								0.134	
	Rewards dimension of subjective orgasm experience	1.44	0.57	0.40	0.28, 2.60	2.53	0.016		1.000

*B, non-standardized beta; SE, standard error; β, standardized beta; 95% CI, 95% confidence interval; R2, adjusted R-squared value; VIF, variance inflation factor.*

For men, a significant model (*F*_(1, 34)_ = 6.41, *p* < 0.05) was obtained in which genital response was significantly related to the rewards dimension of the subjective orgasm experience in masturbation (*β* = 0.40, *p* < 0.05). This variable explained 13.4% of the variance in genital response.

## 4 Discussion

Providing validity evidence in people who engage in same-sex relationships was proposed for the masturbation parameters (i.e., negative attitudes, solitary sexual desire, current frequency and subjective orgasm experience) by relating them to elicited sexual arousal in a laboratory context (i.e., genital response, and ratings of both sexual arousal and genital sensations) and controlling for the effect of propensity for sexual excitation.

First, gender differences were observed in some masturbation parameters. Compared to women, men showed more negative attitudes toward masturbation, more frequently engaged in masturbation and experienced orgasm more intensely on its rewards dimension. Previous studies have indicated that men report more negative attitudes toward masturbation than women (Blanc, 2024; Sierra et al., 2023) but, in contrast, they report a higher frequency for this behavior (Driemeyer et al., 2017; Leistner et al., 2023; Sierra et al., 2023). The higher masturbation frequency in men could be attributed to the sexual double standard, which would grant men greater sexual freedom or permissiveness than women (Álvarez-Muelas et al., 2021). It is known that masturbation in men plays a compensatory role for low frequency or dissatisfaction in sexual relationships (Regnerus et al., 2017; Sierra et al., 2023), which could explain this binomial of negative attitudes toward masturbation and higher frequency observed in men because men consider it a “second-class” sexual behavior. For intensity of the subjective orgasm experience, the literature usually indicates that women report greater intensity in the masturbation context *vs.* in sexual relationships (Cervilla and Sierra, 2022; Sierra et al., 2023). However, in a similar laboratory context to that in the present study, Cervilla et al. (2023) found that men scored higher on the same dimension of orgasm. Compared to women, men consume more pornography when masturbating (Sun et al., 2016), tend to instrumentalize orgasm in masturbation (Mangas et al., 2024a) and their orgasmic experience would be related more to physical aspects (Salisbury and Fisher, 2013). Together, they could explain the relation between the intensity of the genital response recorded in the laboratory to images of explicit sexual content and the rewards dimension of orgasm.

The results indicated for both women and men that the only parameter of masturbation associated with sexual arousal experienced in response to films of people of the same sex masturbating was the subjective orgasm experience.

In women, significant positive correlations were observed among the affective, sensory and intimacy dimensions of the subjective orgasm experience in masturbation and the rating of sexual arousal. The intensity of the subjective orgasm experience was the only masturbation parameter to be associated with sexual arousal experienced in response to explicit sexual stimuli (subjective sexual arousal in this case). In women, the fact that greater intensity in the affective, sensory, and intimacy experience of orgasm was related to more subjective sexual arousal was not at all strange because women are generally those who experience orgasm more intensely in masturbation (Cervilla and Sierra, 2022; Muñoz-García et al., 2023; Sierra et al., 2023). It should be noted that the affective aspects of subjective orgasm experience become important in women (Rowland et al., 2019; Sierra et al., 2021, 2023). This association suggests a transfer between solitary sexual activity and sexual relationships, a fact that has been recently noted by Pérez-Amorós et al. (2024). However, when all the masturbation parameters were introduced into the explanation of the rating of sexual arousal, only the intimacy dimension played a significant role, which left out the affective and sensory dimensions from the explanatory model, along with solitary sexual desire, which explained 24.1% of the variance of the rating of sexual arousal. The salience of the intimacy dimension could be related to women’s greater need for sexual intimacy (Greeff and Malherbe, 2001; Shrier and Blood, 2015). Furthermore, women’s solitary sexual desire has been associated with the pursuit of immediate rewards, such as sexual arousal (Cervilla et al., 2023; Dosch et al., 2016). This could explain why both the intimacy dimension and solitary sexual desire are relevant for explaining the rating of sexual arousal.

In men, the affective and rewards dimensions of subjective orgasm experience were positively associated with genital response (i.e., penile circumference). Moreover, the rewards dimension was the only variable with explanatory capacity for genital response and explained 13.4% of its variance. Previous studies have highlighted the relevance of the rewards dimension of subjective orgasm experience in the masturbation context (Cervilla et al., 2024b; Mangas et al., 2024a; Muñoz-García et al., 2023). In line with this, Cervilla et al. (2024b) observed this dimension’s explanatory capacity on the propensity for sexual excitation in men. As previously mentioned, men’s tendency to instrumentalize orgasm (Mangas et al., 2024b) could be one of the reasons that lie behind this greater salience of the rewards dimension, along with traditional sexual roles, i.e., with men seeking more physical pleasure (Masters et al., 2013). This result supports the finding observed in other studies showing that greater intensity of the subjective orgasm experience in masturbation is associated with better sexual functioning (Cervilla and Sierra, 2022; Cervilla et al., 2024b).

The results indicated that the examined masturbation parameters explained the objective sexual arousal measure (i.e., penile circumference) in men, whereas the explained sexual arousal was subjective in women. Previous laboratory studies have reported the more marked relevance of the psychophysiological measure of genital response in men (Arcos-Romero et al., 2019; Mangas et al., 2024b and the rating of sexual arousal in women (Arcos-Romero et al., 2019; Cervilla et al., 2024b). These differences could be due to sexual arousal being linked more with physical aspects in men and with psychological aspects in women (Arcos-Romero et al., 2019; Basson, 2000; Granados et al., 2017). Thus, we can affirm that the role that the parameters of masturbation have in the understanding of sexual arousal presents different nuances between men and women. In men, the rewards dimension of subjective orgasm experience in masturbation plays an important role in explaining genital response. In women, the intimacy dimension of orgasm experience in masturbation and solitary sexual desire become important for explaining the rating of sexual arousal. These data highlight not only the need for detailed assessment of masturbation behavior for use as a therapeutic tool, but also the need to consider gender differences in the development of sex education programs.

In both men and women, the only masturbation parameter associated with sexual arousal was subjective orgasm experience, to which solitary sexual desire was added only for women. That is, of the four examined parameters (i.e., negative attitudes toward masturbation, solitary sexual desire, current masturbation frequency, subjective orgasm experience), only two presented validity evidence in the present laboratory study. Sexual pleasure, feeling aroused and learning about one’s own body are the main reasons for masturbation in both men and women (Herbenick et al., 2023; Regnerus et al., 2017). Masturbation is characterized by self-focus, autonomy and control (Foust et al., 2022; Goldey et al., 2016). Thus greater control and autonomy during masturbation, and exploring one’s own body, could explain the ability to connect more profoundly with one’s sensations, such as orgasm intensity. So they play an important role in explaining sexual arousal. Given the implications of masturbation behavior for sexual health (Cervilla et al., 2024a), these results highlight the importance of evaluating this behavior in detail. Particular attention should be paid to the intimacy dimension of orgasm and solitary sexual desire in women, and the rewards dimension in men. However, in women *vs.* men, the fact that more masturbation parameters were observed to be involved in explaining sexual arousal is consistent with previous studies, where the need for more variables to explain sexuality dimensions, such as orgasm, comes over (Arcos-Romero and Sierra, 2020; Cervilla and Sierra, 2022).

We should highlight the lack of relevance of the negative attitude toward masturbation in this study as it did not present any relationship with sexual arousal. In general, attitudes toward sexuality have evolved toward more positive perspectives, especially in young people such as those in this study (Arcos-Romero et al., 2024; Vallejo-Medina et al., 2014), which may explain why negative attitudes toward masturbation have less weight in explaining variables such as sexual arousal. For example, although orgasm satisfaction has been shown to be related to negative attitudes toward masturbation, this relationship is weak compared to other variables (Sierra et al., 2021). Furthermore, this study examined only the direct association between negative attitude toward masturbation and sexual arousal. Future studies should consider this attitude as a potential mediating or moderating variable.

This study has some limitations. The sample consisted of young, healthy and cisgender university students, which limits the generalizability of its results to the general population. This is due to the artificial nature of laboratory studies, which prioritize internal validity and limit external validity. Future studies should include older people, men and women with sexual dysfunction, and other gender identities. Future studies could address this issue by including a more diverse and representative sample, including participants of different ages, socioeconomic backgrounds, individuals with different health conditions (e.g., diabetics), and individuals with a broader range of gender identities. To achive this, we could use more inclusive sampling techniques, such as stratified random sampling, to ensure that different subgroups are adequately represented. Finally, we should bear in mind that volunteers in laboratory studies on sexuality may have different psychosexual characteristics from the general population (Arcos-Romero et al., 2024), which could bias the generalizability of the results.

## 5 Conclusion

The results of this study provide validity evidence for some solitary masturbation parameters in people who engage in same-sex relationships: solitary sexual desire and subjective orgasm experience. An association is observed between masturbation behavior and sexual arousal experienced in a specific situation, e.g., when exposed to explicit sexual stimuli. This highlights the importance of considering masturbation to be relevant behavior for sexual health while considering some gender-specific nuances. In men, the rewards dimension of subjective orgasm experience in masturbation plays an important role in explaining genital response. In women, the intimacy dimension of orgasm experience in masturbation and solitary sexual desire become important for explaining the rating of sexual arousal. Although a first impression might indicate that the percentage of sexual arousal variance explained by masturbation parameters is small (24.1% in women and 13.4% in men), if we consider that any dimension of sexuality is determined by the interaction of biological, psychological and social variables, we can conclude that this is an acceptable percentage. Future studies should consider other variables (e.g., hormone levels). This study provides a profounder understanding of solitary masturbation, sexual behavior that is often relegated to the background. This research also focuses on sexual diversity populations, for whom sexuality research is limited. The results emphasize the importance of considering solitary masturbation when assessing and treating sexual problems. Furthermore, these findings highlight the need for further research on masturbation, as it is a behavior that has historically been associated with stigma and guilt, especially among women (Carvalheira and Leal, 2013). It is critical to deepen our understanding of this behavior, as it can potentially serve as a valuable therapeutic tool for sexual wellness.

## Data Availability

The datasets presented in this study can be found in online repositories. The names of the repository/repositories and accession number(s) can be found at: Figshare: https://doi.org/10.6084/m9.figshare.27682086.v1.
